# Investigating Structural Dynamics of KCNE3 in Different Membrane Environments Using Molecular Dynamics Simulations

**DOI:** 10.3390/membranes12050469

**Published:** 2022-04-27

**Authors:** Isaac K. Asare, Alberto Perez Galende, Andres Bastidas Garcia, Mateo Fernandez Cruz, Anna Clara Miranda Moura, Conner C. Campbell, Matthew Scheyer, John Paul Alao, Steve Alston, Andrea N. Kravats, Charles R. Sanders, Gary A. Lorigan, Indra D. Sahu

**Affiliations:** 1 Natural Science Division, Campbellsville University, Campbellsville, KY 42718, USA; ikasar294@students.campbellsville.edu (I.K.A.); apere15@students.campbellsville.edu (A.P.G.); andresbastidas4556@students.campbellsville.edu (A.B.G.); mfern512@students.campbellsville.edu (M.F.C.); ammour424@students.campbellsville.edu (A.C.M.M.); cccamp92@students.campbellsville.edu (C.C.C.); mwsche97@students.campbellsville.edu (M.S.); salston@campbellsville.edu (S.A.); 2 Department of Chemistry and Biochemistry, Miami University, Oxford, OH 45056, USA; alaooj@miamioh.edu (J.P.A.); kravatan@miamioh.edu (A.N.K.); 3 Center for Structural Biology, Department of Biochemistry, Vanderbilt University, Nashville, TN 37232, USA; chuck.sanders@vanderbilt.edu

**Keywords:** KCNE3, structural dynamics, lipid bilayers, molecular dynamics simulation, membrane mimetic

## Abstract

KCNE3 is a potassium channel accessory transmembrane protein that regulates the function of various voltage-gated potassium channels such as KCNQ1. KCNE3 plays an important role in the recycling of potassium ion by binding with KCNQ1. KCNE3 can be found in the small intestine, colon, and in the human heart. Despite its biological significance, there is little information on the structural dynamics of KCNE3 in native-like membrane environments. Molecular dynamics (MD) simulations are a widely used as a tool to study the conformational dynamics and interactions of proteins with lipid membranes. In this study, we have utilized all-atom molecular dynamics simulations to characterize the molecular motions and the interactions of KCNE3 in a bilayer composed of: a mixture of POPC and POPG lipids (3:1), POPC alone, and DMPC alone. Our MD simulation results suggested that the transmembrane domain (TMD) of KCNE3 is less flexible and more stable when compared to the N- and C-termini of KCNE3 in all three membrane environments. The conformational flexibility of N- and C-termini varies across these three lipid environments. The MD simulation results further suggested that the TMD of KCNE3 spans the membrane width, having residue A69 close to the center of the lipid bilayers and residues S57 and S82 close to the lipid bilayer membrane surfaces. These results are consistent with previous biophysical studies of KCNE3. The outcomes of these MD simulations will help design biophysical experiments and complement the experimental data obtained on KCNE3 to obtain a more detailed understanding of its structural dynamics in the native membrane environment.

## 1. Introduction

KCNE3 is a potassium channel accessory transmembrane protein belonging to the KCNE family that regulates the function of various voltage-gated potassium channels such as KCNQ1 and KCNQ4 [1,2,3,4]. KCNE3 has been expressed in the small intestine, colon, and human heart [5,6,7]. Previous studies have shown that in the presence of KCNE3, KCNQ1’s voltage sensitivity shows a linear current-voltage (I-V) relationship that gives rise to a potassium ion conductivity in non-excitable cells as polarized epithelial cells of the colon, small intestine, and airways [3,8,9]. Its malfunction has been proven to contribute to health disorders such as cardiac arrhythmia, long QT syndrome, tinnitus, cystic fibrosis, and Menière’s disease [3,5,10,11,12,13,14,15]. For such a biologically significant membrane protein, little information is known about the structural and dynamic properties of KCNE3 in native like membrane environment, where interactions between lipids and proteins help stabilize the structure of the protein and influence protein function within the membrane. Previous NMR studies of KCNE3 in detergent micelles and isotropic bicelles by the Sanders lab have shown KCNE3’s structure consists of an extracellular N-terminus surface associated amphipathic helix connected by a loop to an alpha helical transmembrane domain [16]. A disordered C-terminus is connected to the transmembrane domain by a short juxta membrane helix [16]. Recent studies by Sun et al. using cryo-electron microscopy (Cryo-EM) showed that KCNE3 tucks its single membrane spanning helix against KCNQ1 at a point that appears to keep the voltage sensor in its depolarized confirmation [8]. However, it is not fully understood how these various sections behave structurally and dynamically in various membrane bilayer environments.

Molecular dynamics (MD) simulations serve as a structure biology tool to complement experimental studies in order to study the stability and structural dynamic properties of membrane proteins at an atomic level [17,18,19,20,21]. Here, we use all-atom MD simulations in the course of 105 ns to study stability and structural dynamic properties of KCNE3 in bilayers composed of POPC (1-palmitoyl-2-oleoyl-*sn*-glycero-3-phosphocholine)/POPG (1-palmitoyl-2-oleoyl-*sn*-glycero-3-phospho-(1′-*rac*-glycerol) (sodium salt)) (3:1), POPC alone, and DMPC (1,2-dimyristoyl-sn-glycero-3-phosphocholine) only. The POPC/POPG mixtures, POPC alone and DMPC are widely used lipid systems to mimic biological membrane bilayers for biophysical studies [16,17]. Previous MD simulation studies on similar membrane proteins and other protein systems have suggested that the simulation times of 10–100 ns can provide reliable analysis of protein–detergent and protein–lipid interactions [17,22,23,24]. We have analyzed MD simulation trajectory data to obtain several structural dynamics related parameters such as backbone root mean square deviation (RMSD), root mean square fluctuation (RMSF), lipid bilayer membrane width, Z-distances, total protein–lipid interaction energy, TMD helical tilt angle, and a heat map of the correlation between parameters, results that yield insight into the stability, molecular motion and interaction of KCNE3 in different phospholipid bilayer membranes.

## 2. Methods

### 2.1. Molecular Dynamics Modeling of Wild-Type KCNE3 in Lipid Bilayers

Nanoscale molecular dynamics (NAMD) [25] version 2.14 with the CHARMM36 force field was employed to perform molecular dynamics simulations on a full length KCNE3 (PDB ID: 2M9Z, the original pdb file is available in the Supporting Information of the ref. [16] in lipid bilayers composed of POPC/POPG (3:1), POPC alone, and DMPC alone [26,27,28]). The simulation set up and input files were generated by using CHARMM-GUI [29]. The visual molecular dynamics software (VMD) Xplor version 1.13 [30,31] was used for MD trajectory data analysis. The bilayer, composed of a pre-equilibrated lipid molecules with a ~12,010.5 Å^2^ surface, was built using membrane builder protocol under CHARMM-GUI [29,32]. The total charge of KCNE3 was 2.0 in the simulation. The positively charged amino acid residues were protonated, and negatively charged amino acids were deprotonated. The histidine (HIS) residues were protonated to the neutral form (HSD). The protein was inserted into the membrane and the system was solvated into a TIP3 water box and ionized to add bulk water above and below the membrane and to neutralize the system with KCl using the membrane builder protocol [29,32]. The final assembled system comprised waters, phospholipids, ions and the protein (a total of ~174,071 atoms). Six equilibration steps of each protein-lipid system were performed for 50–200 ps with 2 fs timesteps using the NAMD program with input files generated by CHARMM-GUI [29,32]. The minimization equilibration utilized collective variable restraints to slowly release system motion and facilitate simulation stability. Starting from this equilibrated system, NAMD simulations were carried out for ~105 ns using Langevin dynamics for the three membrane environments [18]. Electrostatic interactions were computed using the Particle-Mesh Ewald algorithm with a 12 Å cutoff distance [33] and Van der Waals interactions were computed with a 12 Å cutoff distance and a switching function to reduce the potential energy function smoothly to zero between 10–12 Å. Periodic-boundary conditions were used, and constant temperature (303 K) and pressure (1 atm) were maintained. Equations of motion were integrated with a timestep of 2 fs and trajectory data was recorded in 20 ps increments [18].

### 2.2. Analysis of the MD Simulation Data

The structures in the MD trajectory data were aligned with respect to the starting structure in the production run for each membrane bilayer environment before further analysis. The stability and structural dynamic behavior of KCNE3 was obtained from the aligned trajectory data by calculating root mean square deviation (RMSD) of all atoms of the backbone, root mean square fluctuation (RMSF), lipid bilayer membrane width, Z-distances, total protein–lipid interaction energy, and TMD helical tilt angle using the scripts available in the VMD software package [30]. The heatmaps for the correlation between different simulation parameters were graphed using Matlab (https://www.mathworks.com accessed on 10 February 2022). The images were prepared using the Igor Pro graphics program (https://www.wavemetrics.com accessed on 10 February 2022). All molecular dynamics simulations were run on the Miami Redhawk cluster computing facility at Miami University.

## 3. Results and Discussions

**Figure 1:**
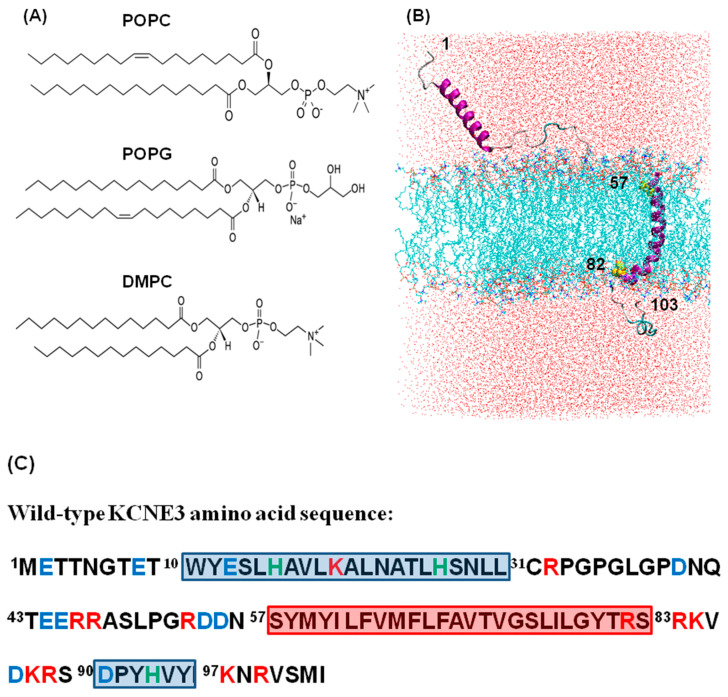
(**A**) Chemical structure of phospholipids used in the NAMD molecular dynamics simulations. (**B**) An illustrative example of the cartoon representation of the NMR structure of KCNE3 (PDB ID: 2M9Z) incorporated into POPC/POPG lipid bilayers and solvated into water box [16]. Amino acid sites 1–56 represent N-terminus, amino acid sites 57–82 represent TMD and sites 83–103 represent C-terminus. The amino acid sites 57 and 82 are colored yellow. (**C**) Amino acid sequence of the wild-type KCNE3 with distribution of charges. Positive charges (Red), negative charges (Blue), and Histidine (Green) are color coded. The highlighted red box represents the transmembrane domain and blue boxes represent N- and C-terminal helices.

The stability and structural dynamic properties of KCNE3 in different phospholipid bilayer environments were investigated using NAMD molecular dynamics simulation trajectory data. A wild-type KCNE3 protein was incorporated into three different lipid bilayer environments including POPC/POPG (3:1), POPC alone, and DMPC alone to study how structural and dynamic properties of KCNE3 behave in different lipid bilayer environments; POPC and POPG are monounsaturated lipids and DMPC is saturated lipid. These lipids are widely used in studying membrane protein/peptides. POPG lipids contain a negative charge and hence the mixture of POPC and POPG at the molar ratio of 3 to 1 may provide more favorable condition to stabilize the TMD of KCNE3 buried into lipid bilayers while spanning the width of the bilayer membrane [34,35]. Figure 1A shows the chemical structure of the lipids used in this study. The protein lipid systems were set up beginning with the NMR structure of KCNE3. KCNE3 was inserted into the respective lipid bilayer with the transmembrane helix spanning the membrane with the C- and N-terminal helices oriented on either side of the bilayer and interacting with the solvent. Figure 1B shows the setup for the KCNE3-POPC/POPG system; the amino acid sequence of the wild-type KCNE3 is indicated in Figure 1C with the red box indicating the transmembrane helix, the blue boxes indicating the N- and C-terminal helices and the distribution of charged amino acids by color codes [16].

### Molecular Motion of KCNE3 in Different Phospholipid Bilayer Environments

**Figure 2:**
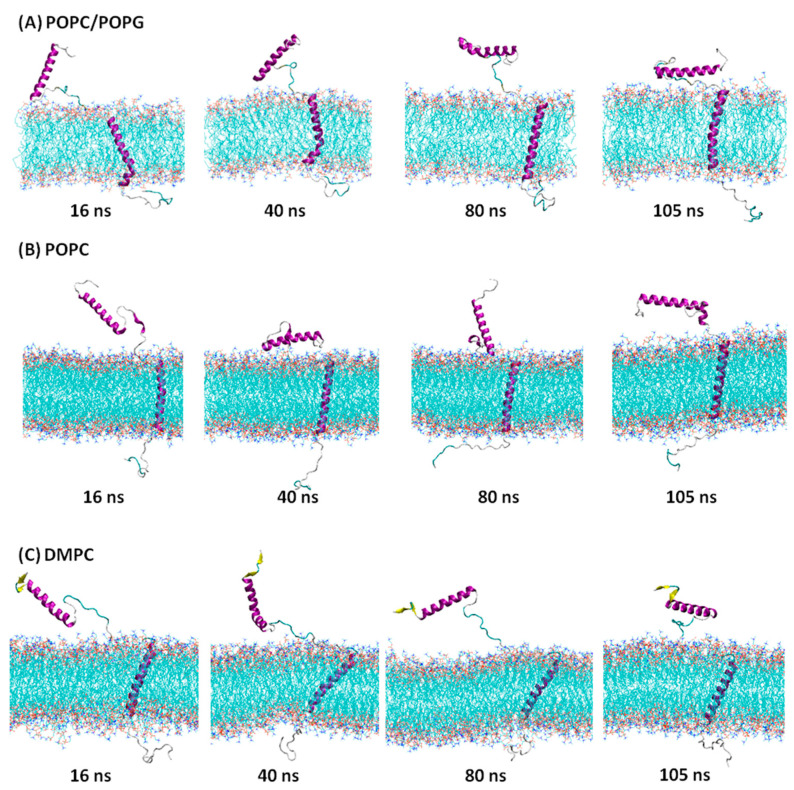
Snapshots of the representative MD simulation trajectory data of KCNE3 at 16 ns, 40 ns, 80 ns, and 105 ns for POPC/POPG (**A**), POPC alone (**B**), and DMPC alone (**C**) lipid bilayers. The hydrogen atom and water are omitted to make visualization simple and clear.

An all-atom molecular dynamics simulation on wild-type KCNE3 in three different lipid bilayer environments was carried out over the course of 105 ns. Figure 2 shows the snapshots of the representative MD simulation output trajectory data of KCNE3 incorporated into all three lipid bilayer systems (POPC/POPG, POPC alone, and DMPC alone) for 16 ns, 40 ns, 80 ns and 105 ns. The interaction of C- and N-termini of KCNE3 with the lipid bilayer surface is flexible and dynamic for all three lipid compositions. Interestingly, the initial few amino acid sites of N-terminal of KCNE3 showed formation of a short beta strand structure in the DMPC lipid system.

**Figure 3:**
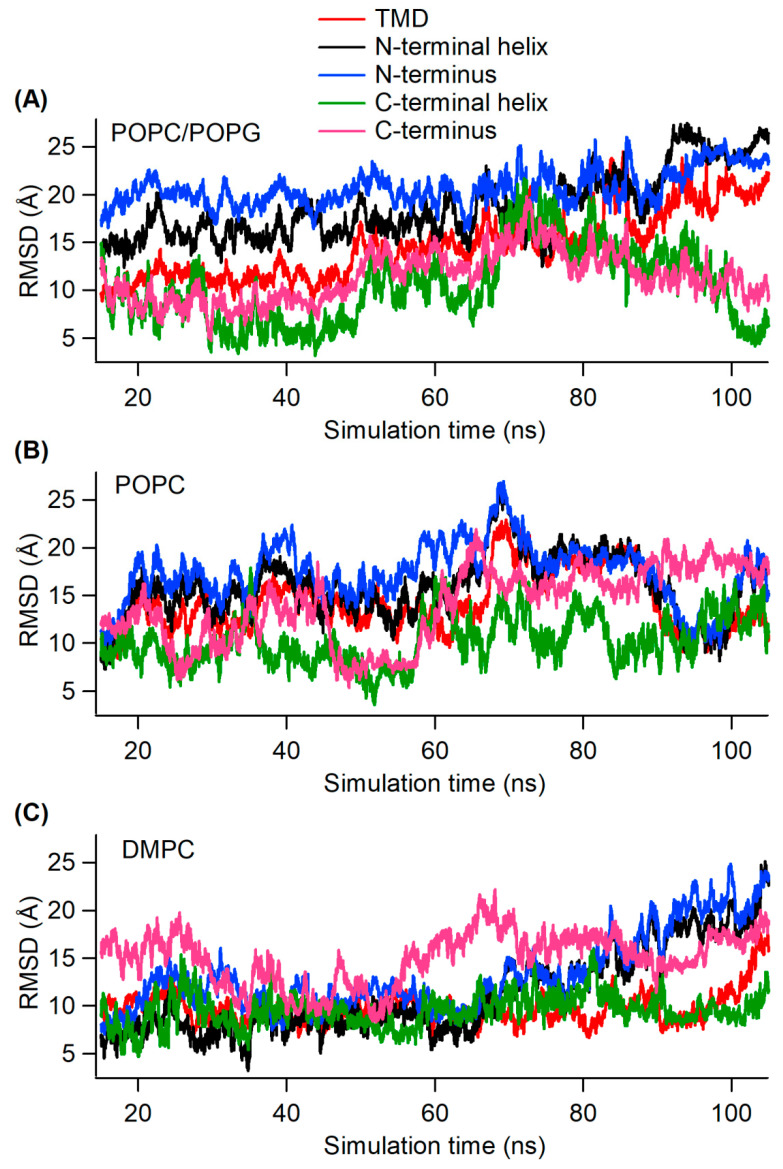
Root mean square deviation (RMSD) as a function of simulation time for different segments of KCNE3 in POPC/POPG (**A**), POPC alone (**B**), and DMPC (**C**).

In order to analyze the conformational stability and molecular motion of the wild-type KCNE3 in membrane environments, a backbone root mean square deviation (RMSD) was calculated from the trajectory data and plotted as a function of simulation time for different segments of the protein including transmembrane domain (TMD), C-terminus, N-terminus, C-terminal helix, and N-terminal helix for POPC/POPG (3:1), POPC alone, and DMPC lipid bilayers as shown in Figure 3. We omitted analysis of the first 15 ns of each trajectory of the production run to avoid the equilibration time of the system. The RMSD measures the mean position of the amino acid residues in the structure of the subsequent simulation frames and compares them to the initial structure [22]. The RMSD is important in identifying regions of the proteins that have higher flexibility as well as regions that are stabilized. The initial trajectories for all simulations in the POPC/POPG and POPC alone systems are similar. The RMSD profile pattern for POPC/POPG (Figure 3A) shows that the RMSD values for the TMD of KCNE3 are lower than that of N-terminus and N-terminal helix until 49 ns and then increases to have similar values by 105 ns. The RMSD values for C-terminal and C-terminal helix are lower than the that of the TMD, N-terminus and N-terminal helix and vary throughout the simulation. Similarly, the RMSD profile pattern for POPC (Figure 3B) shows that the RMSD values for the TMD of KCNE3 are very close to that of N-terminus and N-terminal helix during the simulation. The RMSD values for the C-terminal helix are relatively lower than the TMD, N-terminus and N-terminal helix and C-terminus with fluctuating values. The RMSD values for the C-terminus are also close to these values, but fluctuate throughout the simulation. The RMSD profile pattern for DMPC (Figure 3C) shows that the RMSD values for the TMD, N-terminus, N-terminal helix, C-terminal helix, and C-terminus behave similarly with similar RMSD values. KCNE3 appears to be more stable in DMPC than in POPC/POPG or POPC alone, as the RMSD profiles for each segment are suppressed by comparison. In the POPC/POPG and POPC alone systems, the N-terminus and N-terminal helix have the highest RMSDs of all the segments of KCNE3. These data suggest that these regions of the protein have conformationally higher backbone fluctuations in the KCNE3 structure. This is expected, as the N-terminus contains a larger number of amino acid residues compared to the C-terminus and the TMD [16]. In the POPC/POPG and POPC alone systems, the RMSD values of the TMD begins at higher values than that of the C-terminus and C-terminal helix. However, the C-terminus and C-terminal helix have larger fluctuations as compared to the TMD, suggesting that the C-terminus is more mobile and unstable than the TMD. The overall fluctuations of the C-terminus are, however, lower than that of the N-terminus. The relatively smaller fluctuations observed for the TMD throughout the simulation suggests that it is the most stable segment of the protein and has the greatest stability of all segments studied. In the DMPC membrane mimetic system, the TMD is observed to have similar backbone fluctuations as in the POPC/POPG and POPC alone systems. However, the C-terminus segment starts out with a higher RMSD than that of the N-terminus, in contrast to the other two POPC/POPG and POPC alone systems. Similarly, higher backbone fluctuations for N- and C-termini reveal a similar level of conformational instability in the DMPC bilayer system. The average RMSD values for different segments of the KCNE3 are also calculated for all three lipid systems from the data in Figure 3 and shown in Table 1. The average RMSD values vary between 10.4–23.5 Å for POPC/POPG, 10.3–17.4 Å for POPC alone, and 9.5–15 Å for DMPC. The average RMSD value for the TMD in DMPC is the least value for TMD of all three lipid systems studied. The C-terminal helix has the lowest average RMSD value when compared to different segments of the protein in all three corresponding lipid systems. The standard deviation calculated of the average RMSD data show higher values for the outside regions of the protein compared to the TMD in all three corresponding lipid systems. The RMSD data for different regions of KCNE3 in different lipid bilayer environments suggest that the backbone flexibility for different segments of KCNE3 is different in POPC/POPG, POPC alone, and DMPC bilayer membranes. Our overall RMSD data suggest that the regions of the KCNE3 that is outside the membrane or interact with the surface are more flexible while DMPC lipid system plays a more stabilizing role.

**Figure 4:**
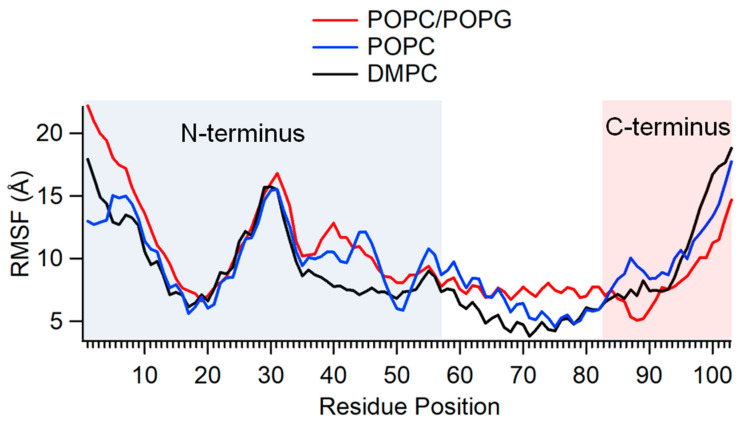
Plot of the root mean square fluctuation (RMSF) of KCNE3 as a function of simulation time for three different lipid compositions: POPC/POPG (Red), POPC (Blue), and DMPC (Black).

The RMSD data only provide the average behavior of the motion of the different segments of the protein while interacting with lipid bilayer membrane. We also wanted to understand how the flexibility of the particular regions assessed above contributed to the overall fluctuations that disturb the KCNE3’s stability. The residue fluctuation profile of bilayer-integrated KCNE3 were quantitatively determined by the root mean square fluctuation (RMSF) throughout the simulation as shown in Figure 4. While the RMSD indicates positional differences of entire structures over the course of the simulation, the RMSF calculates how much a residue fluctuates during the simulation [22]. Consequently, it helps determine the flexibility of individual residues. Figure 4 shows the RMSF for KCNE3 residues in the three bilayer conditions. The profile for KCNE3 is similar for all three bilayer compositions. Overall, residues 1–9 (unstructured region) and ~25–35 (around the terminal of N-terminal helix) of the N-terminus and residues ~96–103 (unstructured region) of the C-terminus have the largest RMSF, suggesting they are the most flexible.

These results agree with the RMSD calculations that highlighted the highest fluctuations in the C- and N-termini. The RMSF of N-terminal residues 11–24 (helical region) and the TMD section from residue 57–82 are lower and indicate stability. The smallest RMSF fluctuations of the TMD region occur in DMPC, which is in agreement with our observations regarding the RMSD of this region. The previous NMR data-restrained molecular dynamics simulation on KCNE3 in DMPC lipid bilayers suggested the dynamic interaction of N- and C-termini helices with membrane surface [16]. These helices contain amphipathic amino acid sequences that do not deeply bury into the lipid bilayers, and hence these helices can dynamically interact with bilayer surfaces. The fluctuation of different segments of KCNE3 as suggested by the RMSF plot is consistent with the RMSD data and earlier NMR studies [16]. Our RMSF data suggest the N- and C-termini are more flexible with higher RMSD values in all three lipid compositions.

**Figure 5:**
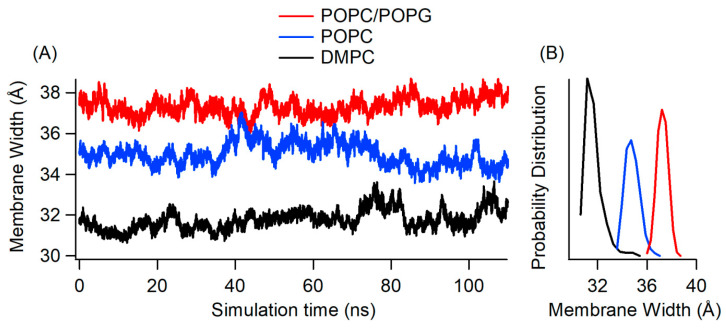
Membrane bilayer width incorporating KCNE3 protein as a function of simulation time (**A**) and membrane width probability distribution (**B**) for POPC/POPG (Red), POPC (Blue), and DMPC (Black) bilayers.

We wanted to better understand the formation of the lipid bilayer in the presence of reconstituted KCNE3, since we observed a suppressed RMSD for the KCNE3 TMD region in DMPC, in comparison to POPC/POPG and POPC alone. Both tails of the DMPC lipid only have 14 carbons, while POPC and POPG have 16 and 18 (Figure 1A). We measured the width of the membrane bilayer as a function of the simulation time for all three membrane mimic environments (POPC/POPG, POPC alone, and DMPC) to determine whether DMPC was forming compacted bilayers that stabilized the KCNE3 TMD. The membrane width was calculated by measuring the distance between the center of mass of the phosphorus of the upper and lower lipid head groups. The membrane width is shown as a time series in Figure 5A, while the probability distribution of the timeseries data is represented in Figure 5B. The membrane width of DMPC is the lowest of all three systems, as expected based on the length of hydrocarbon chains. The membrane width probability distribution plot (Figure 5B) shows the membrane width peak is centered around 37 Å for POPC/POPG, 35 Å for POPC and 31 Å for DMPC. The membrane width for POPC/POPG lipid bilayers is thicker than that of POPC lipid bilayers, despite having the same number of carbon atoms in the acyl chains. 

**Figure 6:**
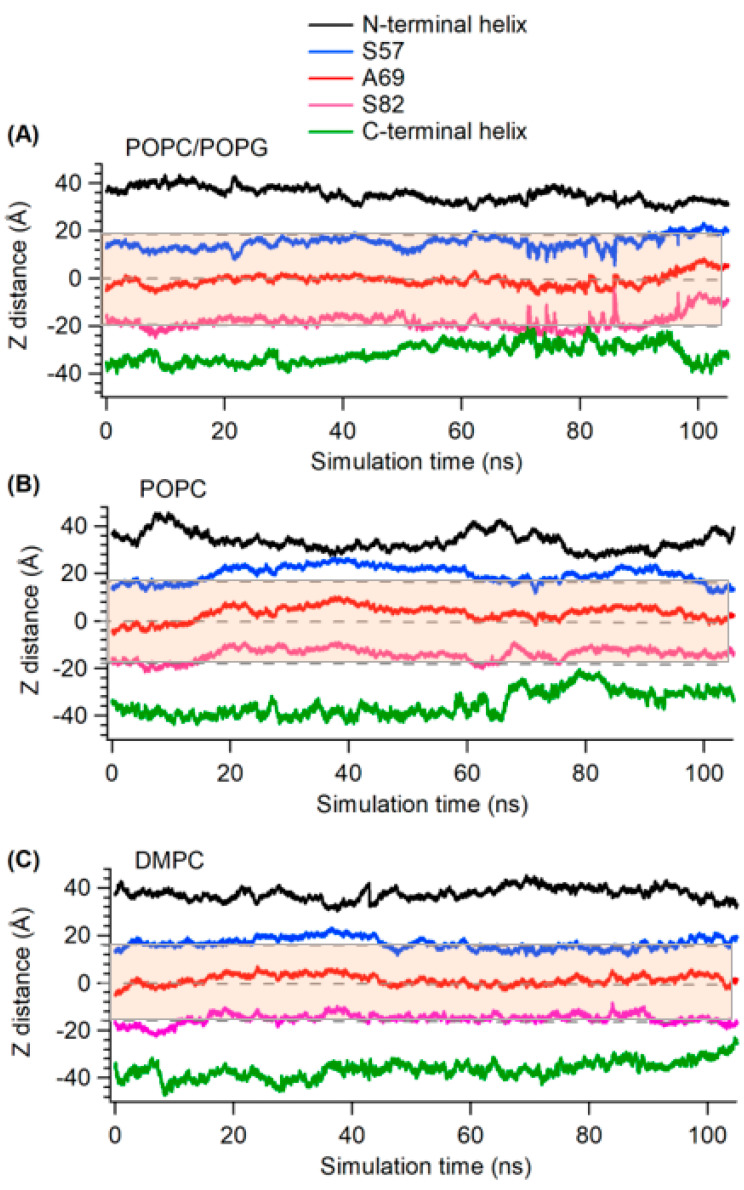
The plot of *z*-axis distance (Z-distance) as a function of simulation time for KCNE3 incorporated into POPC/POPG (**A**), POPC (**B**), and DMPC (**C**) lipid bilayers. Shaded regions represent the average width of the corresponding lipid bilayers calculated from Figure 5.

Next, we wanted to understand the protein topology with respect to the lipid bilayer membrane, since we observed that each bilayer had a different membrane width. The membrane thickness is oriented about the *z*-axis with the center of mass of the membrane bilayer located at Z = 0. We calculated the distance from the *z*-axis (Z-distance) of different segments of KCNE3 from the center of the mass of the lipid bilayers in all three different lipid membrane environments (POPC/POPG, POPC alone and DMPC). Previous NMR studies in micelles and isotropic bicelles suggested that amino acid residue sites 57 to 82 belong to the TMD of the KCNE3 that spans the membrane bilayer width [16]. The Z-distances of the center of mass of the N-terminal helix, residues S57, A69 and S82, and the C-terminal helix from the center of mass of the lipid bilayers were calculated from the MD trajectory data. These Z-distance data can provide us with the information on how much various residues and different segments in the protein structure moved away from the center of the lipid bilayers when incorporated into different membrane environments. Figure 6 shows the plot of Z-distance as a function of simulation time for center of mass of different segments (N- and C-termini helices), and sites S57, A69, and S82 of TMD of KCNE3 in three different lipid bilayer environments (POPC/POPG, POPC alone, and DMPC). Figure 6A indicates that the TMD termini sites S57 and S82 are close to the surface of the lipid bilayer and span the width of the membrane for POPC/POPG lipid bilayers. The amino acid residue A69 lies close to the center of the lipid bilayers for POPC/POPG as indicated by the Z-distance around zero. The Z-distance for N- and C-termini helices varies outside the membrane width range. A similar trend of Z-distance profiles were observed for POPC alone and DMPC lipid bilayer environments. However, the Z-distance ranges for the TMD termini residues S57 and S82 for DMPC is lower than that for POPC/POPG and POPC alone. This is expected, as the DMPC bilayer width is lower than that of the POPC/POPG and POPC alone (Figure 5). The behavior of Z-distance pattern profile for these lipid bilayer environments is consistent with the membrane width profile shown in Figure 5.

**Figure 7:**
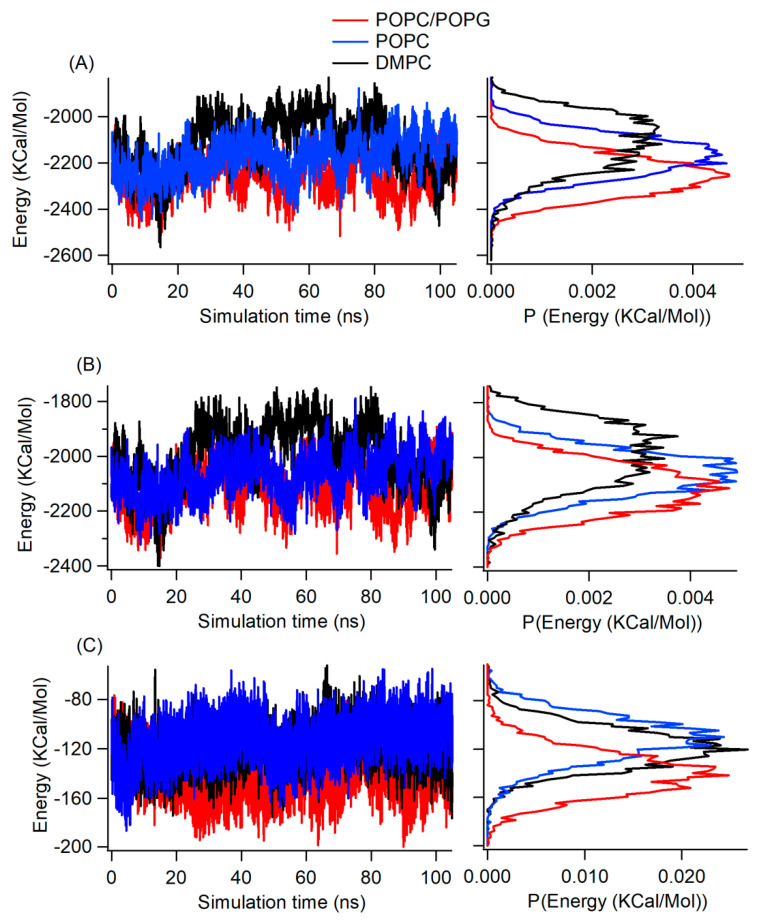
Internal energy of KCNE3 in lipid bilayer membranes as a function of simulation time (left panel) and corresponding histogram (right panel) for total internal energy (**A**), electrostatic energy (**B**) and van der Waals energy (**C**). The *x*-axis of the histogram plot represents the probability distribution.

To understand the stability of the interaction of the KCNE3 reconstituted into lipid bilayer membrane environments, we calculated the internal energy of KCNE3 and plotted this energy as a function of simulation time for all three membrane bilayer environments. The corresponding histograms for total internal energy, electrostatic energy contribution and van der Waals energy contribution are shown in Figure 7. Figure 7A shows similar total energy profiles for all three systems. When the data is represented as a probability distribution (right panel), the total internal energy of the KCNE3 is the lowest, with more favorable values in the POPC/POPG lipid bilayers. The total internal energy of KCNE3 increases for POPC bilayers and is the least favorable in DMPC. Figure 7B shows the similar internal energy trends and histogram profiles for electrostatic energy contribution when compared to the total energy profile for all three systems. Figure 7C shows a lower van der Waals contribution to the total internal energy when compared to the electrostatic energy contribution. The probability distribution (Figure 7C, right panel) shows the van der Waals energy of KCNE3 has a slightly lower value in POPC/POPG lipid bilayers when compared to the POPC alone and DMPC alone systems both having similar van der Waals energy contributions. The electrostatic interactions are the dominant contribution to the total energy for all three lipid environments. The trend of the total internal energy in all three lipid environments suggests that the overall protein structure is more stable in POPC/POPG bilayer membrane compared to the cases of POPC and DMPC. Our hypothesis is that when the KCNE3 is unable to interact with the lipids, it relies on internal interactions to stabilize the structure. 

**Figure 8:**
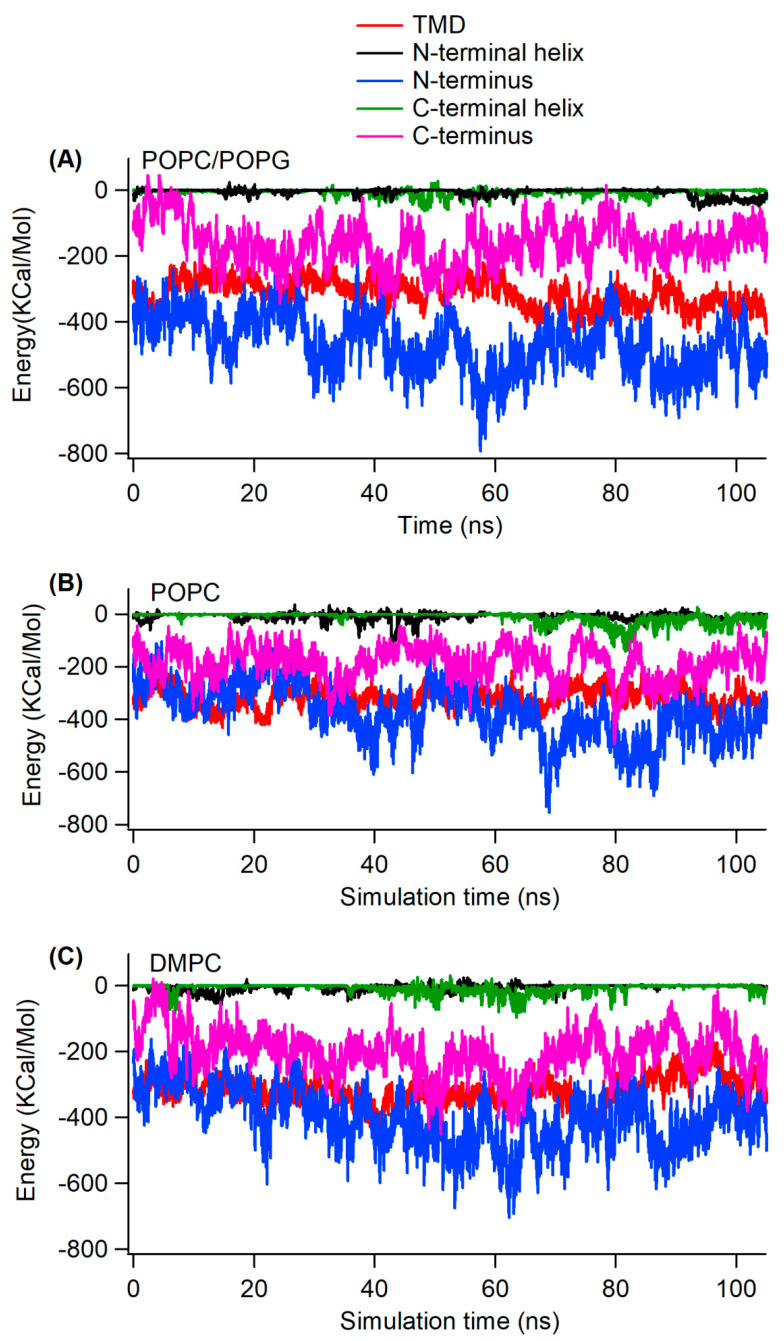
Interaction energy of different segments of KCNE3 with lipid bilayer membranes as a function of simulation time for POPC/POPG (**A**), POPC (**B**), and DMPC (**C**) lipid bilayers.

To test this hypothesis, we computed the interaction energy of different segments of the KCNE3 with the lipid bilayer membrane as shown in Figure 8. In all three lipid environments, the interaction energy of the TMD section with the lipid is lower than that of C-terminus, C-terminal helix, and N-terminal helix with the respective lipid. The interaction energy of the N-terminus is close to interaction energy of the TMD but fluctuates throughout the simulation. While the interactions of the N- and C- termini appear to be strong and exhibit large fluctuations, the N- and C-termini helices weakly interact with the lipid. Inspection of the trajectory data suggests these helices are closely interacting with the membrane surface throughout the simulation, where the interaction energy attains the lowest values. The average interaction energies for each section of KCNE3 during the simulation were calculated for all three lipid systems from the interaction energy data (Figure 8) as shown in Table 2. The average interaction energy for the TMD of KCNE3 is not significantly different for all three lipid systems. Similarly, other segments of the protein have similar average interaction energy (within the error) in all three corresponding lipid systems. However, the standard deviation values are larger for the N- and C-termini in all three lipid systems. These data suggest that the interactions of the N- and C-termini of KCNE3 with the membrane surface are dynamic. 

These interaction energy data suggest that the N-terminus interacts most strongly with the membrane surface but the interaction is dynamic and unstable, as the standard deviation in the average energy calculation is very high for this segment of the protein. While the interaction energy of TMD is higher than that of the N-terminus, the stability of the TMD structure may be attributed to the internal energy of the protein its transient interactions with water molecules [16].

**Figure 9:**
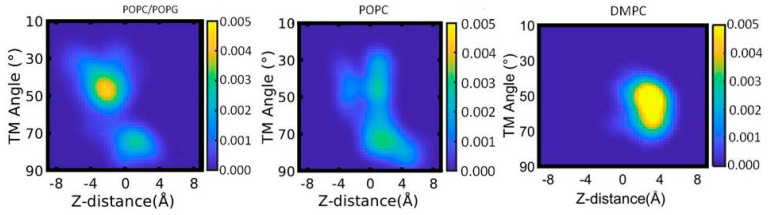
Probability density plot of transmembrane (TM) helical tilt angle against the Z-distance of TMD of KCNE3 in different lipid bilayer membranes. The yellow color indicates the highest probability, and the blue color represents the lowest probability.

To better understand the conformational stability and the interaction of the transmembrane domain (TMD) of KCNE3 with membrane bilayers, we calculated the helical tilt of the TMD within the bilayer. In our previous results, we saw that the membrane thickness was dependent upon the lipid environment, though there were minute differences in the Z-distances of the terminal residues of the TMD helix. We wanted to establish whether the deformation of the helix occurred to accommodate the structure within the bilayer. We have plotted the probability density of the transmembrane (TM) helical tilt of KCNE3 with the membrane normal and the Z-distance of the TMD of KCNE3 from the center of the mass of lipid bilayers for all three different membrane environments (POPC/POPG, POPC alone, and DMPC) as shown in Figure 9. The initial Z-distance fluctuates around 0 Å. When KCNE3 is embedded in the POPC/POPG lipid bilayer, two conformations of the TMD helix are observed; the dominant population is centered around a Z-distance of −3 Å and a helical tilt of 45°, while the second less populated conformation is at a Z-distance of 2 Å and a helical tilt of 75°. In the case of POPC alone, similar conformations are observed as in the POPC/POPG system, though the populations are more diffuse with sampling of many intermediate states between the two dominant conformations. By comparison, only one dominant conformation exists in the DMPC lipid, which is unique to DMPC and not observed in the other two lipid environments. The highest probability is centered around a Z-distance of 3.5 Å, while the helical tilt fluctuates between 45° and 70°. These data suggest that there is a strong correlation between TM helical tilt angle and Z-distance for POPC/POPG bilayers and DMPC bilayers, and a weak correlation for POPC bilayers. Interestingly, the dominant probability density for the DMPC membrane appears for the Z-distance of the TMD of around 4 Å from the center of the mass of the bilayers and TM helical angle of around 45–70°. This suggests that the TMD helix tilts to remain embedded within the lipid bilayers. However, in DMPC the TMD helix is more stationary at the Z-distance and samples less tilt angles, suggesting that it is more stable in this lipid system. In contrast, when the TMD helix is in POPC/POPG and POPC alone, the tilting behavior of the TMD helix results in changes in Z-position, suggesting the TMD helix is more mobile within these lipids. These probability distribution patterns are also consistent with the membrane width data shown in Figure 5. 

**Figure 10:**
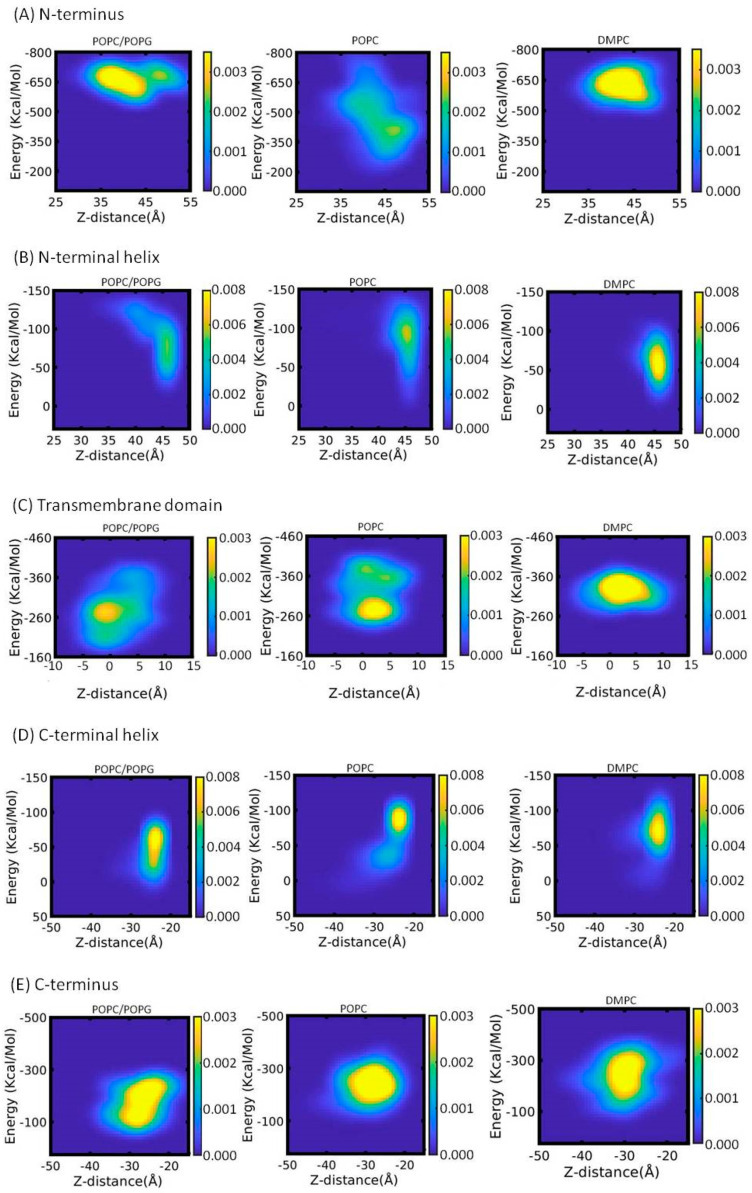
Probability density plot of total interaction energy of N-terminus (**A**), N-terminal helix (**B**), Transmembrane domain (**C**), C-terminal helix (**D**) and C-terminus (**E**) of KCNE3 with lipid bilayers against corresponding Z-distances from the center of mass of the lipid bilayers for different lipid bilayer membranes. The yellow color indicates the highest probability and blue color represents the lowest probability.

We wanted to further understand the conformational stability and interaction of different segments of KCNE3 with membrane bilayers. We plotted the correlation between the total interaction energy of different segments of KCNE3 (N-terminus, N-terminal helix, TMD, C-terminal helix and C-terminus) and the corresponding Z-distances from the center of the mass of the membrane bilayers in Figure 10. Figure 10A shows the probability density for the N-terminus. Similar trends are observed for POPC/POPG and DMPC, with one dominant population that varies between Z-distances of 35–50 Å and interacts strongly with the lipids with energies ranging from −550 to −750 kcal/mol. Interestingly, the probability density for POPC alone is more dispersed and involves much lower interaction energies. Visualization of the trajectory data suggests that the interaction of the N-terminus with the POPC membrane surface is dynamic and very unstable, with a wide spreading of its portion above the surface with occasionally anchoring to it. For the N-terminal helix of KCNE3 (Figure 10B), the probability densities are similar for all three lipid environments. However, an additional disperse density with higher interactions and Z-distances closer to the lipid head groups is observed for POPC/POPG. Visualization of the trajectory data suggests that N-terminal helix also interacts dynamically with the POPC/POPG membrane surface and develops a bending in the helix during the interaction during certain periods of simulation times. A similar trend of a dominant population for the TMD helix is observed for all three systems (Figure 10C). However, an additional dispersed density with higher interactions with same Z-distance has been observed in POPC. The probability density for the TMD helix also shows higher interactions in DMPC. For the C-terminal helix (Figure 10D) and the C-terminus (Figure 10E), similar populations are observed in all three lipid systems with a slightly weaker interactions observed in the POPC/POPG system. Together, these data suggest that the TMD of KCNE3 stably interacts with all three lipid systems, with DMPC conferring the greatest stability. While the N-terminus of KCNE3 is interacting strongly with POPC/POPG, it is more dynamic and less stable. The interaction trend of C-terminus is similar in POPC alone and DMPC. The weak interaction of the C-terminus in POPC/POPG suggests that the unanchored regions of either termini are stabilized by the interactions with water. The probability density pattern for different KCNE3 segments observed in these three environments are consistent with our RMSD, RMSF, membrane width, Z-distances, and total interaction energy data (Figure 3, Figure 4, Figure 5, Figure 6 and Figure 7).

NMR studies by the Sanders lab on KCNE3 in LMPC (lyso-myristoylphosphatidyl choline) micelles and DMPG (dimyristoylphosphatidylglycerol)/ DHPC (dihexanoylphosphatidylcholine) isotropic bicelles have suggested that KCNE3 adopts a single α-helical transmembrane domain (57–81). This is connected to a flexible loop with N-terminal surface associated amphipathic helix (10–30) and a short juxtamembrane helix (90 to 95) and a disordered C-terminus (96 to 103) [16]. The previous double electron electron resonance (DEER) electron paramagnetic resonance (EPR) data on KCNE3 in POPC/POPG bilayered vesicles suggested that the TMD helix of KCNE3 adopts a moderate curvature with residues T71, S74, and G78 facing the concave side of the curvature [16]. The TMD of KCNE3 is crucial to its function, and the curvature is important for binding to the activated-state channel [16]. A recent cryo-electron microscopic (Cryo-EM) spectroscopic study on the KCNE3-KCNQ1 complex in detergent micelles suggested that there is a deviation on the structure of KCNE3 interacting with KCNQ1 relative to the KCNE3 NMR structure model in isotropic bicelles with a root mean square deviation (RMSD) of 7.6 Å between the two structures [8]. Our all-atom molecular dynamics simulation data for 105 ns obtained on KCNE3 in POPC/POPG, POPC alone and DMPC bilayers reported in this study suggested that the center of mass of the KCNE3 TMD is slightly increased and more stable in DMPC when compared to POPC/POPG and POPC alone. In contrast, N- and C-termini were more conformationally flexible and interacted differently in all three environments. The N- and C-termini helices dynamically interacted with the solvent or partially interacted with the membrane surface. The MD simulation results further suggested that the TMD of KCNE3 spans the membrane bilayer width with the amino acid residue A69 situated close to the center of lipid bilayers and the residues S52 and S82 are close to the surface of the membrane bilayer. The total internal energy of KCNE3 suggested that the POPC/POPG lipid bilayer membrane provides more stability in protein–membrane interactions. Our molecular dynamics simulation data are consistent with earlier experimental biophysical studies on KCNE3 [8,16,36]. Extending the MD simulation time longer than 105 ns may provide additional insight on the structural dynamic properties of KCNE3 while interacting with different mimetic systems. 

## 4. Conclusions

All atom molecular dynamics simulations for 105 ns were performed on KCNE3 incorporated into POPC/POPG, POPC alone and DMPC alone membrane bilayer environments to study the structural dynamic properties of KCNE3. The MD simulation results suggested that the TMD of the KCNE3 is less conformationally flexible and more stable when compared to the N- and C-termini in all three membrane environments. The N- and C-termini of KCNE3 are conformationally more flexible and dynamic in all these three lipid environments. The MD simulation results further suggested that the TMD of KCNE3 spans the membrane width, with residue A69 located near the center of the lipid bilayers and residues S57 and S82 located at the opposing lipid bilayer membrane surfaces. These MD simulation results complement the experimental biophysical studies of KCNE3 in lipid bilayer membranes to illuminate its structural dynamic properties in more detail.

## Figures and Tables

**Table 1:** Average RMSD calculated from the RMSDs shown in Figure 3. The error represents standard deviation.

	Average RMSD (Å)		
	POPC/POPG	POPC	DMPC
**TMD**	14.7 ± 3.5	14.2 ± 3.1	9.7 ± 1.6
**N-terminal helix**	18.6 ± 3.5	15.9 ± 3.3	11.3 ± 4.4
**N-terminus**	23.5 ± 3.8	17.4 ± 3.1	13.3 ± 4.0
**C-terminal helix**	10.4 ± 4.0	10.3 ± 2.5	9.5 ± 1.6
**C-terminus**	11.3 ± 2.6	14.0 ± 3.9	15 ± 2.8

**Table 2:** Average interaction energy calculated from the interaction energy shown in Figure 8. The error represents standard deviation.

	Average Interaction Energy (KCal/Mol)		
	POPC/POPG	POPC	DMPC
**TMD**	−313.7 ± 42.3	−316.6 ± 37.5	−317.5 ± 42.2
**N-terminal helix**	−5.6 ± 10.9	−8.0 ± 14.8	−5.4 ± 9.4
**N-terminus**	−465.3 ± 85.3	−371.8 ± 105.3	−401.3 ± 86.8
**C-terminal helix**	−4.9 ± 8.8	−11.6 ± 20.2	−7.5 ± 13.7
**C-terminus**	−162.5 ± 64.5	−184.7 ± 63.7	−200.6 ± 68.5

## Data Availability

Not applicable.

## Abbreviations

MD	molecular dynamics;
RMSD	root mean square deviation;
RMSF	root mean square fluctuation;
LMPC	lyso-myristoylphosphatidyl choline;
DMPC	1,2-dimyristoyl-sn-glycero-3-phosphocholine;
DHPC	dihexanoylphosphatidylcholine;
DMPG	dimyristoylphosphatidylglycerol;
POPC	1-palmitoyl-2-oleoyl-*sn*-glycero-3-phosphocholine;
POPG	1-palmitoyl-2-oleoyl-*sn*-glycero-3-phospho-(1′-*rac*-glycerol) (sodium salt);
TMD	Transmembrane Domain;
EPR	electron paramagnetic resonance;
DEER	double electron-electron resonance.
